# Engaging the CD40-CD40L pathway augments T-helper cell responses and improves control of *Mycobacterium tuberculosis* infection

**DOI:** 10.1371/journal.ppat.1006530

**Published:** 2017-08-02

**Authors:** Jonathan Kevin Sia, Erica Bizzell, Ranjna Madan-Lala, Jyothi Rengarajan

**Affiliations:** 1 Emory Vaccine Center, Emory University, Atlanta, GA, United States of America; 2 Department of Medicine, Division of Infectious Diseases, Emory University School of Medicine, Atlanta, GA, United States of America; New Jersey Medical School, UNITED STATES

## Abstract

*Mycobacterium tuberculosis* (Mtb) impairs dendritic cell (DC) functions and induces suboptimal antigen-specific CD4 T cell immune responses that are poorly protective. Mucosal T-helper cells producing IFN-γ (Th_1_) and IL-17 (Th_17_) are important for protecting against tuberculosis (TB), but the mechanisms by which DCs generate antigen-specific T-helper responses during Mtb infection are not well defined. We previously reported that Mtb impairs CD40 expression on DCs and restricts Th_1_ and Th_17_ responses. We now demonstrate that CD40-dependent costimulation is required to generate IL-17 responses to Mtb. CD40-deficient DCs were unable to induce antigen-specific IL-17 responses after Mtb infection despite the production of Th_17_-polarizing innate cytokines. Disrupting the interaction between CD40 on DCs and its ligand CD40L on antigen-specific CD4 T cells, genetically or via antibody blockade, significantly reduced antigen-specific IL-17 responses. Importantly, engaging CD40 on DCs with a multimeric CD40 agonist (CD40LT) enhanced antigen-specific IL-17 generation in *ex vivo* DC-T cell co-culture assays. Further, intratracheal instillation of Mtb-infected DCs treated with CD40LT significantly augmented antigen-specific Th_17_ responses *in vivo* in the lungs and lung-draining lymph nodes of mice. Finally, we show that boosting CD40-CD40L interactions promoted balanced Th_1/_Th_17_ responses in a setting of mucosal DC transfer, and conferred enhanced control of lung bacterial burdens following aerosol challenge with Mtb. Our results demonstrate that CD40 costimulation by DCs plays an important role in generating antigen-specific Th_17_ cells and targeting the CD40-CD40L pathway represents a novel strategy to improve adaptive immunity to TB.

## Author summary

Tuberculosis (TB) remains a serious global health problem and understanding how to induce protective immunity to *M*. *tuberculosis* (Mtb) remains a major challenge. While antigen-specific CD4 T cells and IFN-γ are important for controlling Mtb infection, they are not sufficient for protecting against TB. We need insights into host pathways that can be targeted to overcome suboptimal antigen-specific immunity induced by Mtb. Dendritic cells (DCs) are antigen presenting cells that orchestrate the adaptive immune response to infection, but Mtb subverts DC-T cell interactions. Therefore, improving the crosstalk between DCs and T cells during Mtb infection has the potential to enhance anti-mycobacterial immunity. Here we identify interaction between CD40 on DCs and CD40L on T cells as a critical mechanism for generating lung Th_17_ cells. By engaging CD40 on DCs using a multimeric reagent, we significantly augmented early Mtb-specific Th_17_ responses in lungs. Intratracheal DC instillation in conjunction with CD40 engagement provided a balanced Th_1_/Th_17_ response and improved control of bacterial burden after aerosol challenge with Mtb. Our studies show that the CD40-CD40L pathway is important for the generation of Mtb-specific Th_17_ responses and targeting CD40-CD40L interactions is a promising avenue for improving adaptive immunity to TB.

## Introduction

Critical to the success of *Mycobacterium tuberculosis* (Mtb) as a pathogen is its ability to manipulate host innate and adaptive immune responses to its benefit. Despite the development of antigen-specific T cell responses following infection, Mtb is able to persist within the host, indicating that Mtb-specific T cell immunity is suboptimal and ineffective at eliminating the pathogen [1, 2]. Indeed, several studies have shown that mice infected with Mtb exhibit delayed initiation of antigen-specific CD4 T cell responses, which is preceded by delayed migration of Mtb-containing dendritic cells (DCs) from the lung to draining lymph nodes [3–5]. Moreover, although IFN-γ and T-helper 1 (Th_1_) responses are important for controlling infection, they are not sufficient to eradicate bacteria and do not protect against developing tuberculosis (TB) [6–8]. Recently, IL-17 and Th_17_ responses have emerged as important for protective immunity to TB [9–16]. Studies in mice suggest that early induction of IL-17 in the lung promotes control of mycobacterial growth, and balanced Th_1_/Th_17_ responses in the lung have been reported to be more effective [17–19]. We previously reported that an avirulent *hip1* (Hydrolase important for pathogenesis 1; Rv2224c) mutant Mtb strain induced significantly higher IL-17 and IFN-γ responses compared to infection with wild type Mtb due to enhanced functions of infected DCs [20, 21]. Together, these studies suggest that wild type Mtb subverts DCs to prevent optimal T-helper responses and that augmenting DC functions during infection may be beneficial for improving protective immunity. While several studies have reported that Mtb manipulation of DC functions leads to suboptimal Th_1_ responses [21–24], we lack insights into Th_17_ generation during Mtb infection. To gain insight into host pathways involved in generating Th_17_ responses during Mtb infection, we sought to define the molecular mechanisms underlying Th_17_ responses following Mtb infection of DCs.

As the primary antigen-presenting cells in the immune system, DCs are instrumental in shaping adaptive immunity and determining the types of antigen-specific T-helper subsets that are generated in response to infection. Upon phagocytosis of the pathogen, DCs present pathogen-derived antigens to naïve CD4 T cells, provide critical costimulatory signals and produce cytokines; these signals initiate antigen-specific T-helper cell activation and polarization towards specific subsets [25–27]. However, beyond the role of cytokines such as IL-1β, IL-6, and IL-23 in polarizing and committing antigen-specific CD4 T cells towards a Th_17_ phenotype, the DC-T cell interactions underlying Th_17_ polarization during Mtb infection are poorly defined. We previously showed that eliminating Hip1-dependent immune evasion mechanisms in Mtb enhanced the capacity of DCs to induce Th_17_ responses and was accompanied by significantly higher expression of the costimulatory molecule, CD40, on infected DCs [21]. Because costimulation of naïve T cells in the context of cognate interactions between DCs and T cells is critical for optimal activation and differentiation of antigen-specific T cells, these data suggested that impaired CD40-dependent costimulation during wild type Mtb infection may lead to suboptimal Th_17_ responses in TB. CD40 has previously been implicated in generating Th_1_ responses during Mtb infection [28], but its role in the polarization of the Th_17_ subset during infection is not defined. We therefore sought to investigate the contribution of the CD40 costimulatory pathway in Th_17_ generation during Mtb infection and determine the effects of augmenting CD40 costimulation on bacterial control. In this study, we show that CD40 expression on DCs is required for the generation of IL-17 responses to Mtb infection and that interaction between CD40 on DCs and CD40L on CD4 T cells is critical for generating antigen-specific IL-17 responses. Importantly, we found that engaging CD40 on DCs via crosslinking with a multimeric CD40 agonist reagent (CD40LT) significantly enhanced antigen-specific IL-17 responses to Mtb. Further, intratracheal instillation of Mtb-infected DCs treated with CD40LT led to significant enhancement of antigen-specific Th_17_ responses in the lungs and mediastinal lymph nodes (MLN) of mice, showing that engaging the CD40-CD40L pathway can overcome suboptimal Th_17_ responses to Mtb *in vivo*. Finally, we show that CD40 engagement in the setting of a DC transfer model enhances control of Mtb following aerosol challenge. Our results demonstrate that the CD40-CD40L pathway is critical for generating IL-17 responses and that targeting this costimulatory pathway represents a novel strategy to potentially improve protection against TB.

## Results

### CD40 on DCs is required for the generation of antigen-specific IL-17 responses during Mtb infection

**Fig 1:**
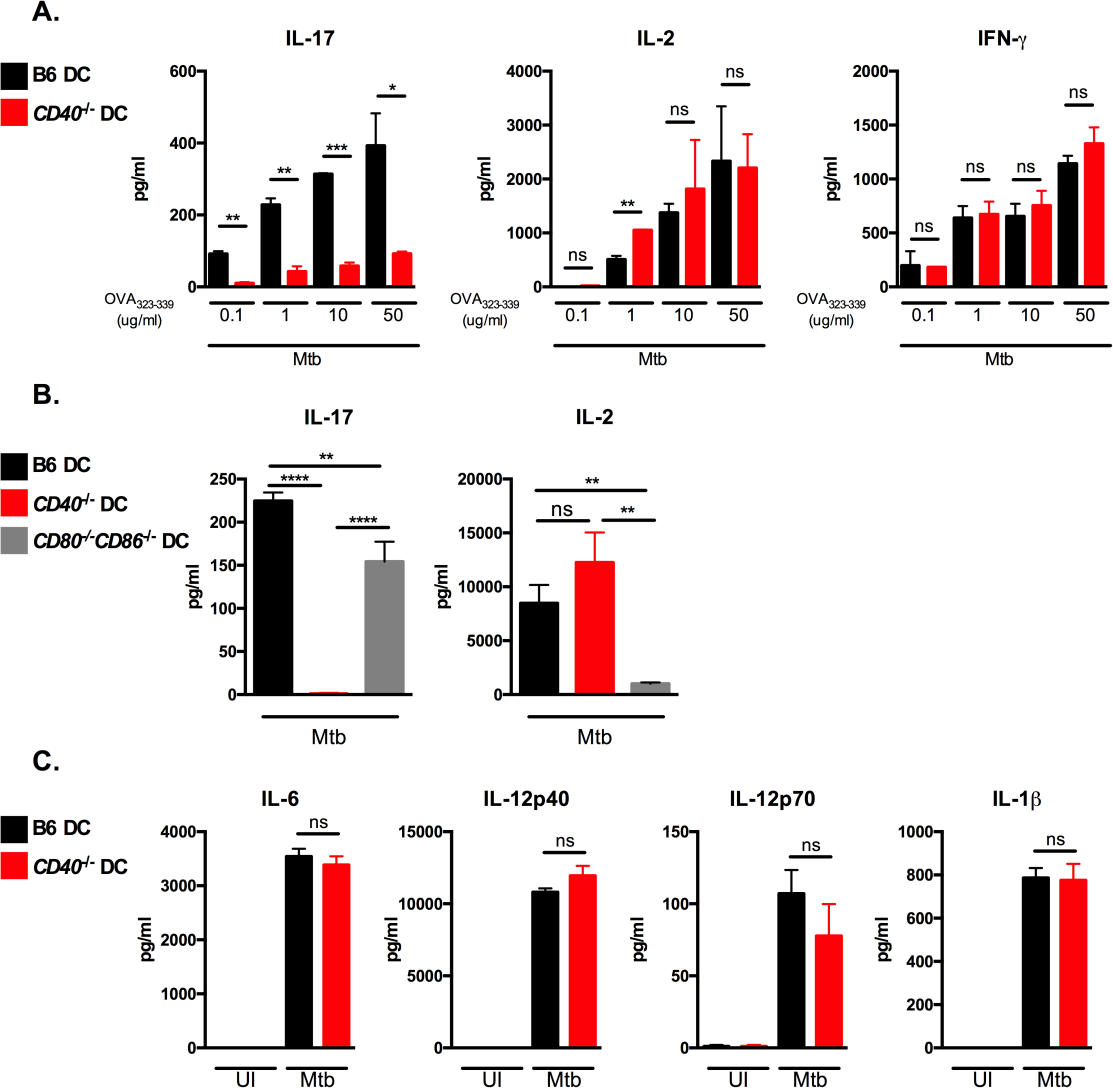
CD40 on DCs is required for the generation of antigen-specific IL-17 responses during Mtb infection. (A) DCs derived from B6 or *CD40*^*-/-*^ mice were pulsed with OVA_323-339_ at the indicated concentrations and infected with Mtb for 24 hours followed by co-culture with purified OT-II TCR-Tg CD4 T cells for 72 hours. Supernatants were assayed for the indicated cytokines by ELISA. (B) DCs from B6, *CD40*^*-/-*^, or *CD80*^*-/-*^*CD86*^*-/-*^ mice were pulsed with 10 μg/ml OVA_323-339_, infected with Mtb and co-cultured with OT-II TCR-Tg CD4 T cells. Cell-free supernatants were harvested after 72 hours and assessed for the indicated cytokines by ELISA. (C) B6 or *CD40*^*-/-*^ DCs were left uninfected (UI) or infected with Mtb. After 24 hours, cell-free supernatants were collected and assessed for the indicated cytokines by ELISA. Data are representative of 3–4 independent experiments. Values are presented as mean ± SD. Statistical significance was determined using a 2-tailed unpaired T-test. * p<0.05; ** p<0.005, *** p<0.0005, **** p<0.0001, ns = not significant.

To test whether CD40 expression is required for differentiation of naïve CD4 T cells into IL-17-producing cells in response to Mtb infection, we used DC-T cell co-culture assays as previously described [21]. We infected bone marrow derived DCs from wild type C57BL/6 mice (B6) or from *CD40*^*-/-*^ mice for 24 hours, followed by co-culture with purified naïve TCR-transgenic (Tg) CD4 T cells isolated from OT-II mice in the presence of OVA_323–339_ peptide (Fig 1A). Supernatants were harvested 72 hours after co-culture and assayed for IL-17, IL-2 and IFN-γ by ELISA. Mtb-infected DCs from B6 mice induced increasing levels of IL-17, IL-2 and IFN-γ cytokines with increasing concentrations of peptide. In contrast, *CD40*^*-/-*^ DCs were significantly impaired in their ability to induce IL-17-producing cells in response to Mtb, but retained the capacity to induce IFN-γ and IL-2 (Fig 1A). These data indicate that CD40 is specifically required to generate antigen-specific IL-17 responses.

To assess whether the defect in IL-17 production was specific for CD40 deficiency, we examined the contribution of the costimulatory molecules CD80 and CD86, which are known to be essential for IL-2 production and are required for optimal T cell proliferation [29, 30]. While DCs that were doubly-deficient in CD80 and CD86 were severely impaired in IL-2 production, their ability to induce antigen-specific IL-17 responses were comparable to DCs from B6 mice (Fig 1B) and did not exhibit the defective IL-17 responses observed in *CD40*^*-/-*^ DCs. These data indicate that CD40-dependent costimulation plays an essential and specific role in the generation of IL-17 responses to Mtb.

Cytokines produced by infected DCs are known to be critical for polarizing antigen-specific CD4 T cell subsets [17, 31]. Since IL-6, IL-1β, and TGF-β have been shown to induce Th_17_ polarization, we sought to assess whether defective IL-17 responses seen in Mtb-infected *CD40*^*-/-*^ DCs was due to defects in their ability to produce innate cytokines following Mtb infection. However, levels of IL-6, IL-1β, and IL-12 produced by DCs from *CD40*^*-/-*^ mice were comparable to the levels seen in DCs from B6 mice (Fig 1C) and bioactive TGF-β was undetectable in all culture conditions. Thus, the inability of *CD40*^*-/-*^ DCs to induce IL-17 responses is not due to impaired innate cytokine responses, suggesting that interaction of CD40 expressed on DCs with its ligand, CD40L (CD154), may be necessary for production of IL-17 by CD4 T cells following Mtb infection.

### CD40-CD40L interaction is critical for inducing antigen-specific IL-17 responses to Mtb infection

**Fig 2:**
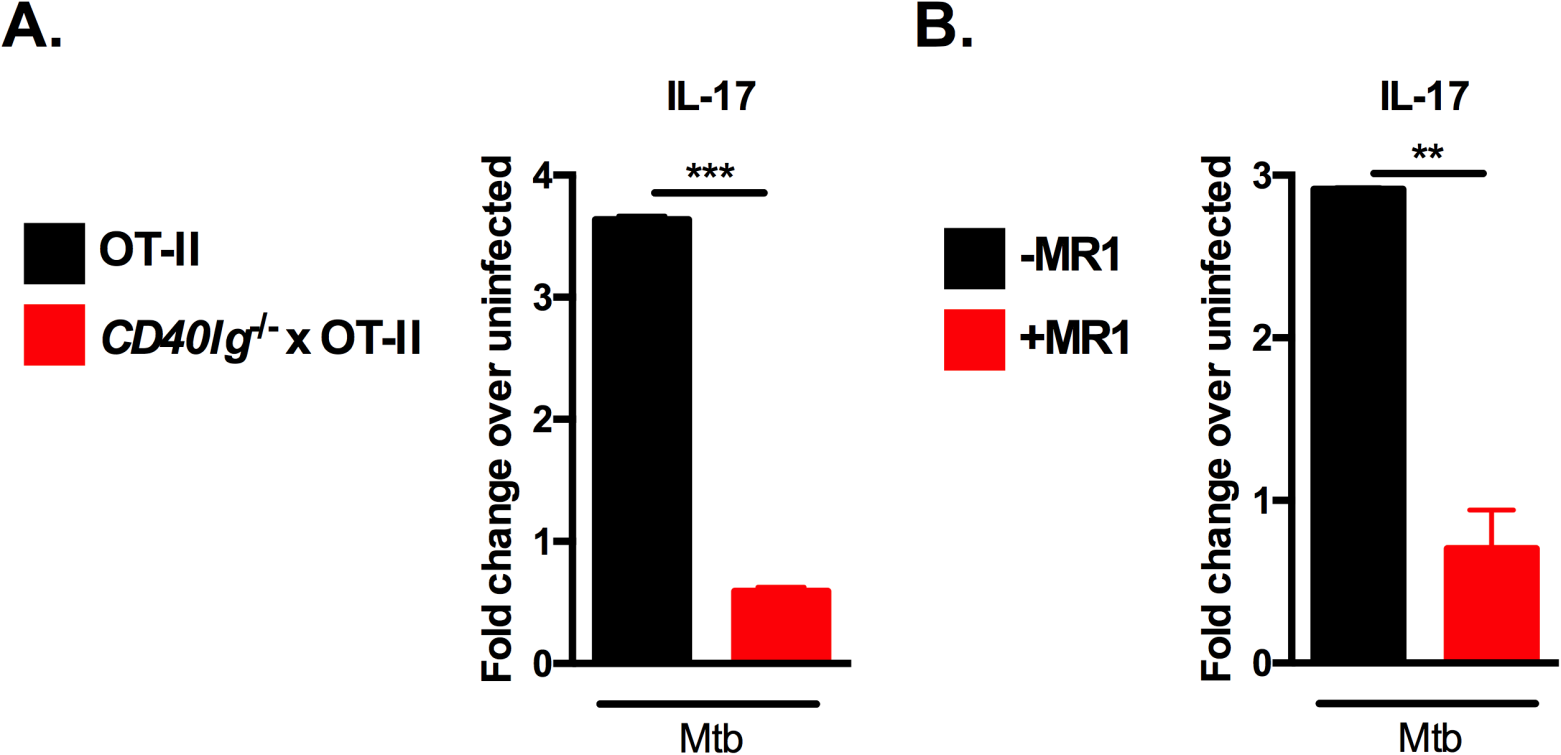
CD40-CD40L interaction is critical for inducing antigen-specific IL-17 responses to Mtb infection. (A) B6 DCs were pulsed with OVA_323-339_ at 10 μg/ml and infected with Mtb for 24 hours followed by co-culture with purified CD4 T cells from OT-II or *CD40lg*^-/-^ x OT-II TCR-Tg mice or (B) with purified OT-II TCR-Tg T cells in the presence or absence of 20 μg/ml anti-CD40L blocking antibody (clone MR1). Cell-free supernatants were collected after 72 hours and IL-17 assessed by ELISA. Data are representative of two independent experiments. Values are presented as mean fold change over uninfected ± SD. Statistical significance was determined using a 2-tailed unpaired T-test. ** p<0.005, *** p<0.0005.

CD40L is expressed on antigen-activated T cells and binding of CD40 with CD40L provides accessory costimulatory signals that are necessary for optimal activation and differentiation of antigen-specific T cells. In order to determine whether interaction of CD40 with CD40L was required for IL-17 generation, we carried out co-culture assays using antigen-specific CD4 T cells isolated from OT-II mice crossed to mice lacking CD40L (*CD40lg*^*-/-*^ x OT-II). This allowed us to test whether CD40L on T cells was required for IL-17 generation in a setting where CD40 expression on DCs remained intact. We found that *CD40lg*^*-/-*^ CD4 T cells were attenuated in their ability to generate IL-17 responses after co-culture with Mtb-infected DCs (Fig 2A), concordant with the defective IL-17 response seen with *CD40*^*-/-*^ DCs (Fig 1). Interestingly, *CD40lg*^*-/-*^ T cells also displayed attenuated IFN-γ and IL-2 responses (S1 Fig), which suggests that lack of CD40L leads to broader defects in T cell responses compared to the absence of CD40. These results show that both CD40 and CD40L are required for optimal IL-17 generation. To further extend our genetic knockouts studies, we carried out co-culture assays in which we blocked CD40-CD40L interactions using saturating doses of a non-agonistic, anti-CD40L monoclonal antibody (clone MR1). This antibody has been shown to successfully block CD40-CD40L interactions *in vitro* (S2 Fig) and *in vivo* [32]. Blockade of CD40-CD40L interaction between Mtb-infected DCs and CD40L-replete antigen-specific CD4 T cells significantly reduced antigen-specific IL-17 responses (Fig 2B). Together, these data show that interaction between CD40 and CD40L is critical for production of IL-17 by CD4 T cells during Mtb infection.

### Engaging CD40 on DCs enhances antigen-specific IL-17 responses

**Fig 3:**
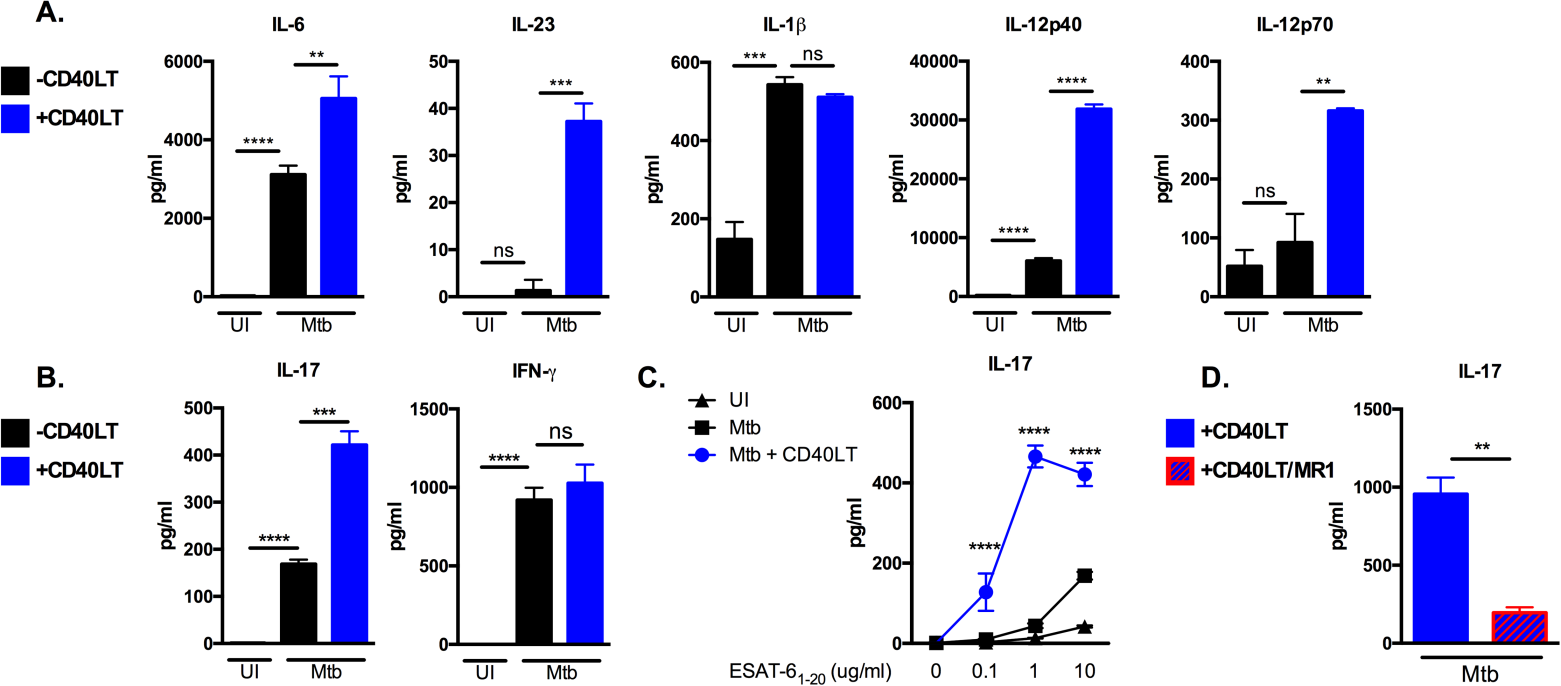
Engaging CD40 on DCs enhances antigen-specific IL-17 responses. (A) B6 DCs were left uninfected or infected with Mtb in the presence or absence of 1 μg/ml multimeric CD40LT reagent (CD40LT) for 24 hours. Cell-free supernatants were collected after 24 hours and the indicated innate cytokines were measured by ELISA. (B) DCs from (A) were pulsed with ESAT-6_1−20_ at 10 μg/ml in the presence or absence of CD40LT and co-cultured with ESAT-6 TCR-Tg T cells for 72 hours. Supernatants were assayed for IL-17 and IFN-γ by ELISA. (C) B6 DCs were pulsed with increasing concentrations of ESAT-6_1−20_ peptide (0, 0.1, 1.0 and 10 μg/ml) either left uninfected (UI) or infected with Mtb in the presence or absence of 1 μg/ml CD40LT for 24 hours followed by co-culture with purified ESAT-6 TCR-Tg CD4 T cells for 72 hours. Supernatants were assayed for IL-17 by ELISA. (D) B6 DCs were pulsed with ESAT-6_1−20_ peptide at 10 μg/ml and infected with Mtb in the presence or absence of 1 μg/ml CD40LT for 24 hours. Co-culture with ESAT-6 TCR-Tg CD4 T cells was done in the presence or absence of 20 μg/ml anti-CD40L blocking antibody (clone MR1). Cell-free supernatants were collected after 72 hours and IL-17 levels determined by ELISA. Data are representative of 3–4 independent experiments. Values are presented as mean ± SD. Statistical significance was determined using a 2-tailed unpaired T-test. ** p<0.005, *** p<0.0005, **** p<0.0001, ns = not significant.

The requirement for the CD40-CD40L pathway in IL-17 generation suggested that boosting interactions between CD40 and CD40L could serve as a tool to augment IL-17 responses. To exogenously engage CD40 on Mtb-infected DCs, we utilized a multimeric CD40 agonist in which two trimeric CD40L constructs are artificially linked (CD40L trimers; CD40LT). The CD40LT reagent effectively aggregates and activates CD40 independently of T cells. Addition of CD40LT to Mtb-infected B6 DCs produced enhanced levels of IL-12 (Fig 3A), consistent with previous reports showing IL-12 induction after CD40 engagement [33]. Importantly, treatment with CD40LT significantly enhanced production of IL-6 and IL-23, which are key cytokines for Th_17_ polarization and expansion (Fig 3A). IL-1β, which also promotes Th_17_ differentiation in combination with IL-6 and IL-23, was not altered by treatment with CD40LT (Fig 3A). Moreover, CD40LT-treated Mtb-infected DCs induced significantly higher levels of IL-17 from co-cultured ESAT-6 TCR-Tg CD4 T cells compared to Mtb-infected DCs lacking CD40 engagement (Fig 3B). In contrast, CD40LT-treatment did not alter production of IFN-γ from antigen-specific CD4 T cells *in vitro* (Fig 3B). These data show that CD40 engagement augments antigen-specific IL-17 generation.

Costimulatory signals synergize with antigen-specific signals downstream of T cell receptor (TCR) ligation to promote full activation of T cells. Absence of signaling through the CD80/86-CD28 costimulatory pathway, for example, results in suboptimal T cell activation and anergic responses [29, 30, 34, 35]. CD28 signaling is thought to function by lowering the T cell activation threshold, thus facilitating optimal T cell activation and IL-2 production. To investigate whether CD40 engagement on DCs similarly impacts the activation threshold of Mtb-specific T cells and whether this, in turn, influences IL-17 production, Mtb-infected DCs were either treated with CD40LT or left untreated, pulsed with increasing concentrations of ESAT-6_1–20_ peptide, and co-cultured with ESAT-6 TCR-Tg CD4 T cells. We found that CD40LT-treated DCs displayed an enhanced capacity to induce IL-17 responses at all antigen doses compared to untreated conditions (Fig 3C). The ability of Mtb-infected DCs to induce IL-17 at lower concentrations of peptide after CD40LT treatment suggests that signals induced by CD40 engagement lowers the threshold for antigen-specific production of IL-17. Thus, CD40-dependent costimulation may serve to overcome suboptimal generation of IL-17 responses elicited early in Mtb infection when antigen levels are low.

In order to dissect the relative contributions of Th_17_-polarizing cytokines and CD40-CD40L interaction on IL-17 responses, Mtb-infected DCs were treated with or without CD40LT and then co-cultured with ESAT-6 TCR-Tg CD4 T cells in the presence of the MR1 CD40L-blocking antibody as described in Fig 2B. Interestingly, antibody blockade of CD40-CD40L interaction significantly decreased antigen-specific IL-17 responses even in the presence of CD40LT (Fig 3D). These data suggest that exogenous engagement of CD40 on DCs that results in enhanced production of Th_17_-polarizing cytokines is not sufficient for generating antigen-specific IL-17 responses in a setting where CD40 cannot interact with CD40L on antigen-specific CD4 T cells.

### CD40 engagement of Mtb-infected DCs induces antigen-specific Th_17_ *in vivo*

Induction of early IL-17 responses on mucosal surfaces of the lung is thought to be important for immunity to Mtb and inducing balanced Mtb-specific Th_1_/Th_17_ responses may enhance protective immunity. To determine whether CD40 engagement on DCs can enhance the induction of Mtb-specific lung Th_17_ responses *in vivo*, we utilized a mucosal transfer approach via intratracheal instillation of DCs. This approach allows for targeted manipulation of Mtb-infected DCs without potential confounding from off-target effects such as CD40 engagement of alveolar macrophages. Transferred DCs have been shown to prime adoptively transferred Mtb-antigen-specific T cells in lymph nodes and lungs of mice by 3 days post-intratracheal instillation [23].

**Fig 4:**
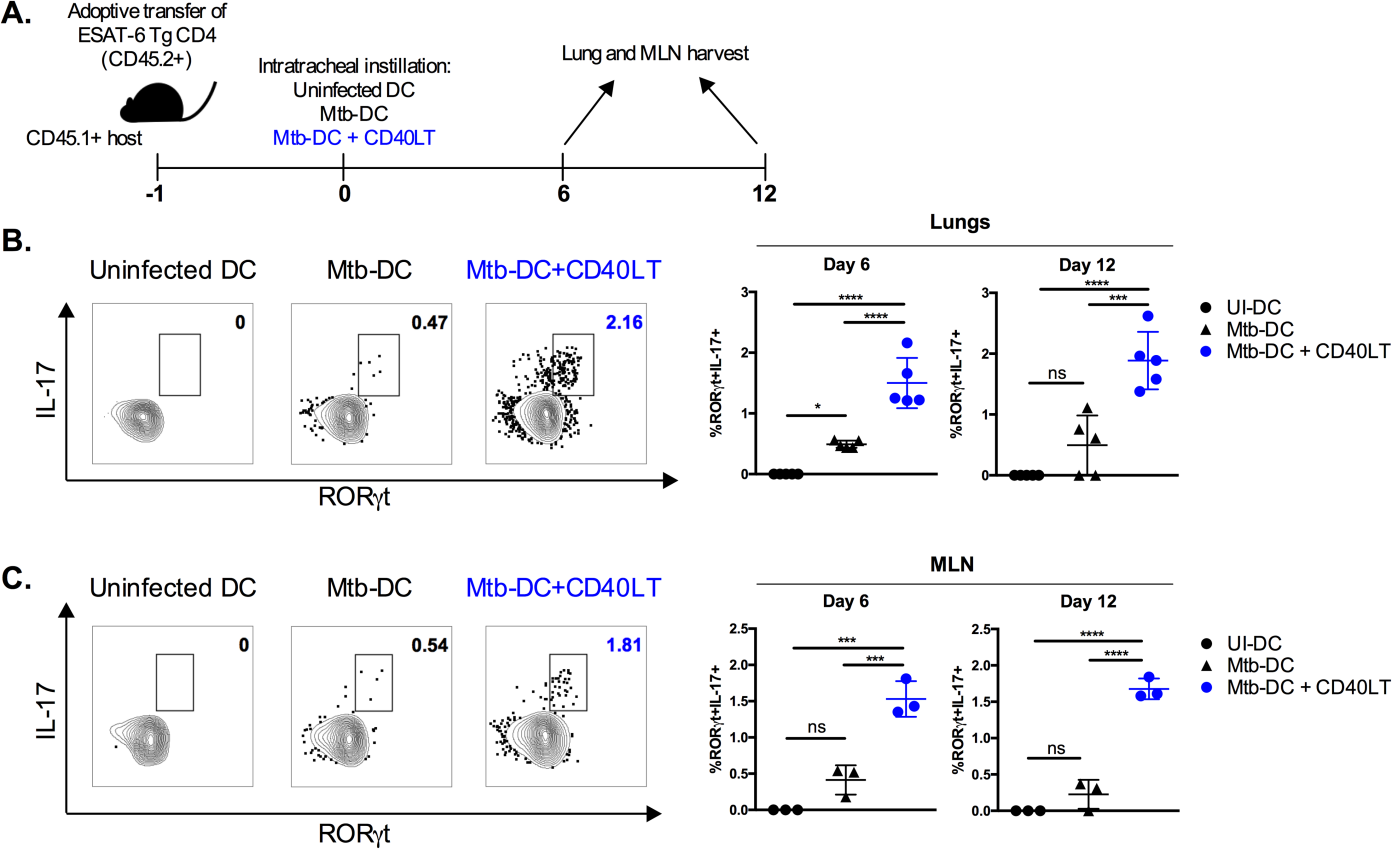
CD40 engagement of Mtb-infected DCs induces antigen-specific Th_17_ *in vivo*. (A) Diagram of experimental design. CD45.2^+^ ESAT-6 TCR Tg CD4 T cells were purified from spleen and lymph nodes and intravenously transferred (1x10^6^ per mouse) into congenic (CD45.1^+^) hosts. Animals were rested for 1 day after which DCs (1x10^6^ per condition) were transferred into recipient hosts by intratracheal instillation: uninfected DCs (UI-DC), Mtb-infected DCs (Mtb-DC) or Mtb-infected DCs with CD40L trimer treatment (Mtb-DC + CD40LT). Lungs and MLN were harvested 6 and 12 days post-intratracheal instillation and CD4 T cell responses assessed. MLN were pooled to attain sufficient cells for restimulation. Representative flow plots (left; day 6 values) and summary graph (right) of the lung (B) and pooled MLN (C) frequencies of RORγt^+^IL-17^+^ cells after ESAT-6_1−20_ restimulation 6 and 12 days after DC transfer. Populations shown have been pre-gated on live CD3^+^CD8^-^γδ TCR^-^CD45.2^+^ singlets. 5 mice were used for each group. Statistical significance was determined using one-way analysis of variance (ANOVA) correcting for multiple comparisons. * p<0.05, *** p<0.0005, **** p<0.0001, ns = not significant.

We adoptively transferred naïve CD45.2^+^ ESAT-6 TCR-Tg CD4 T cells into CD45.1^+^ congenic hosts. The next day, we transferred DCs infected with Mtb in the presence or absence of CD40LT by intratracheal instillation (Fig 4A). At 6 and 12 days after DC transfer, we assessed Th_17_ responses in the lungs and MLN by determining the expression of IL-17 and RORγt in CD45.2^+^ ESAT-6-specific CD4 T cells by intracellular cytokine staining (ICS) and flow cytometry. Engaging CD40 on Mtb-infected DCs using CD40LT enhanced the frequency of ESAT-6-specific RORγt^+^IL-17^+^ T cells in the lungs (Fig 4B) and MLN (Fig 4C). Notably, the majority of IL-17^+^ cells expressed RORγt, the transcription factor that determines Th_17_ lineage commitment [36], indicating that CD40LT-treated Mtb-infected DCs polarized CD4 T cells into Th_17_ cells.

### Enhanced antigen-specific Th_17_ responses in the lungs and MLN of mice following transfer of CD40LT-engaged Mtb-infected DCs or *hip1* mutant Mtb-infected DCs

**Fig 5:**
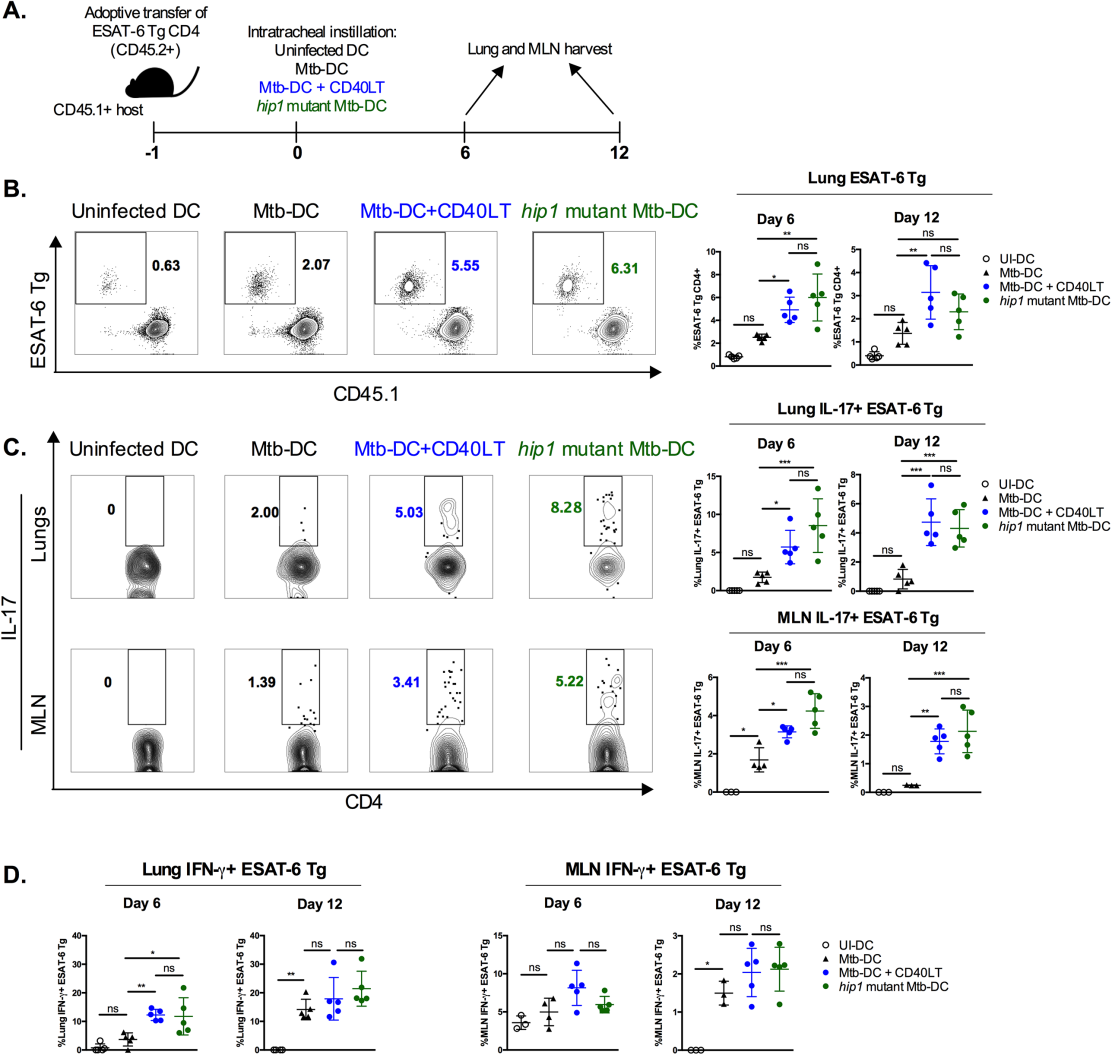
Enhanced antigen-specific Th_17_ responses in the lungs and MLN of mice following transfer of CD40LT-engaged Mtb-infected DCs or *hip1* mutant Mtb-infected DCs. (A) Diagram of experimental design. As before, purified CD45.2^+^ ESAT-6 TCR Tg CD4 T cells were adoptively transferred 1 day before intratracheal instillation of DCs: uninfected DCs (UI-DC), Mtb-infected DCs (Mtb-DC), Mtb-infected DCs with CD40L trimer treatment (Mtb-DC + CD40LT), or *hip1* mutant Mtb-infected DC (*hip1* mutant-DC). Lungs and MLN were harvested 6 and 12 days post-intratracheal instillation and CD4 T cell responses assessed. (B) Representative flow plots (left; day 6 values) and summary graph (right) of the frequencies of CD45.2^+^ ESAT-6 TCR-Tg CD4 T cells in the lungs 6 and 12 days post-instillation. (C) Representative flow plots (left; day 6 values) and summary graphs (right) of the frequencies of IL-17^+^ ESAT-6 TCR-Tg CD4 T cells in the lungs (top) and MLN (bottom) after stimulation with ESAT-6_1−20_ peptide (10 μg/ml). (D) Summary graphs of the frequencies of IFN-γ^+^ ESAT-6 TCR-Tg CD4 T cells in the lungs (left) and MLN (right) after ESAT-6_1−20_ restimulation (10 μg/ml). Populations shown have been pre-gated on live CD3^+^CD8^-^γδ TCR^-^CD45.2^+^ singlets. 5 mice were used for each group. Statistical significance was determined using one-way analysis of variance (ANOVA) correcting for multiple comparisons. * p<0.05; ** p<0.005, *** p<0.0005, ns = not significant.

To determine Th_1_ and Th_17_ responses in the lungs and MLN of mice at 6 and 12 days after intratracheal instillation of DCs, we assessed IFN-γ and IL-17 production in CD45.2^+^ ESAT-6 TCR-Tg T cells by flow cytometry (Fig 5A). Transfer of Mtb-infected DCs that were treated with CD40LT resulted in a greater expansion of ESAT-6 TCR-Tg CD4 T cells compared to Mtb DCs that did not receive exogenous CD40LT, and was comparable to the expansion of ESAT-6 TCR-Tg CD4 T cells in response to *hip1* mutant Mtb-infected DCs (Fig 5B). Moreover, transfer of CD40LT-treated, Mtb-infected DCs significantly enhanced the frequencies of antigen-specific Th_17_ cells in lungs and MLN compared to Mtb-infected DCs alone and was comparable to the Th_17_ frequencies elicited by *hip1* mutant Mtb-infected DCs (Fig 5C). We also observed higher frequencies of antigen-specific IFN-γ^+^ CD4 T cells in the lung, but not MLN, on day 6 post-intratracheal transfer of either Mtb-infected CD40LT-treated DCs or *hip1* mutant Mtb-infected DCs compared to their untreated counterpart (Fig 5D). 12 days after intratracheal instillation of DCs, CD45.2^+^ ESAT-6-specific IFN-γ responses in the lungs were comparable, suggesting that DCs that did not receive CD40LT were delayed in inducing Th_1_ responses relative to Mtb-infected CD40LT-treated and *hip1* mutant Mtb-infected DCs. Interestingly, antigen-specific CD4 T cells producing IL-17 and IFN-γ were mutually exclusive populations and double producing cells were not detected. These data demonstrate that engagement of the CD40 pathway can overcome deficits in Th_17_ generation during Mtb infection and leads to enhanced antigen-specific Th_1_ and Th_17_ responses *in vivo*.

### CD40 engagement of DCs enhances control of Mtb infection

**Fig 6:**
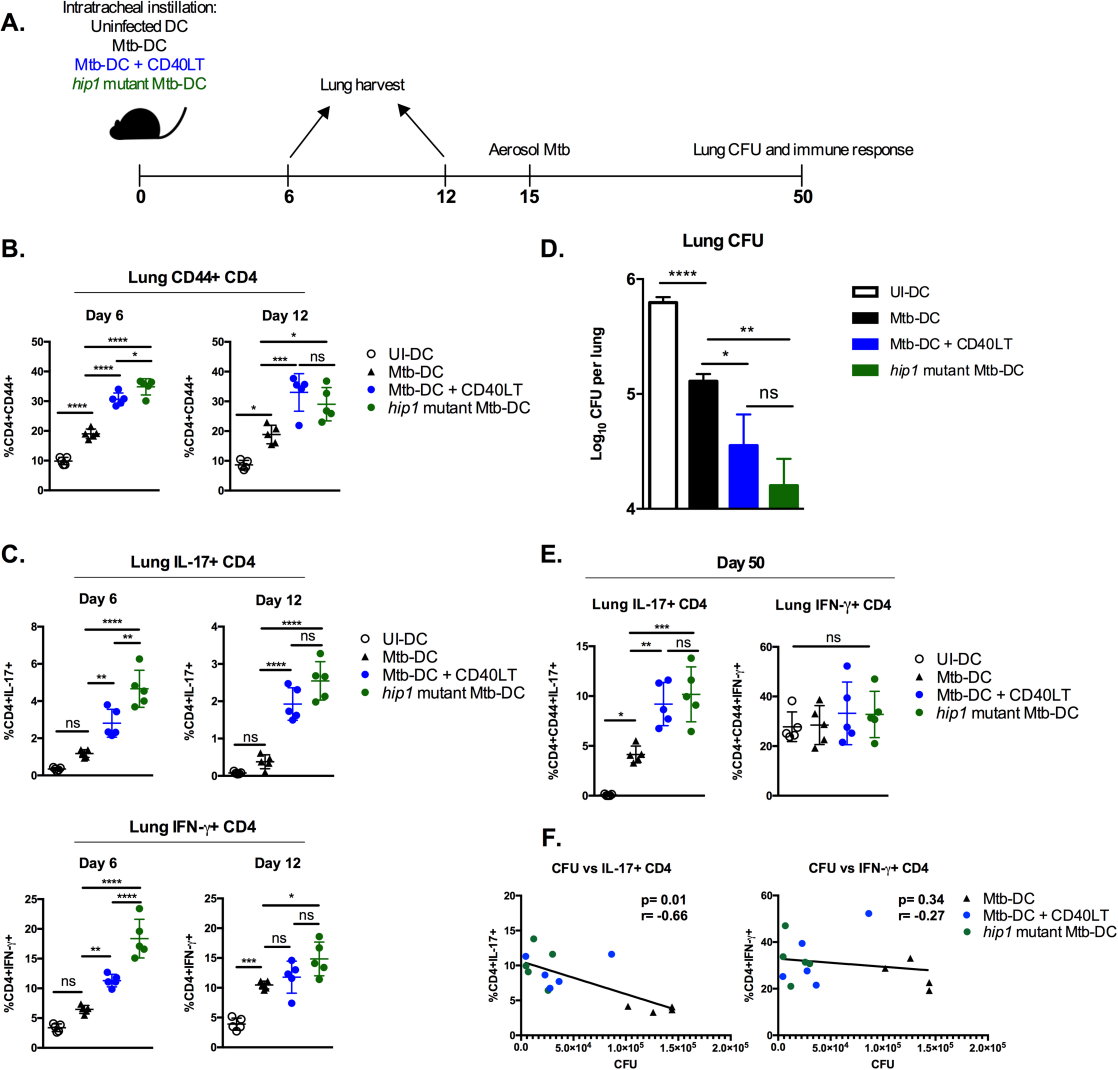
CD40 engagement of DCs enhances control of Mtb infection. B6 DCs were left uninfected or infected with heat-killed Mtb in the presence or absence of CD40LT treatment (1 μg/ml), or infected with heat-killed *hip1* mutant Mtb, each for 24 hours. Cells were washed twice and reconstituted in PBS to deliver 1x10^6^ DC per mouse intratracheally. (A) Diagram of experimental design. Lung responses were assessed 6 and 12 days post-instillation. Mice were infected through the aerosol route with ~100 CFU Mtb 15 days post-instillation and bacterial burden was assessed 35 days (5 weeks) post-challenge. (B) Frequencies of CD44^+^ CD4 T cells in the lungs 6 and 12 days after intratracheal instillation of DCs. (C) Frequencies of IL-17^+^ (top) and IFN-γ^+^ (bottom) CD4^+^ cells in the lungs 6 and 12 days after intratracheal instillation of DCs. Cells were stimulated with PMA (80 ng/ml) and ionomycin (500 ng/ml). (D) Lung bacterial burden 35 days post-challenge (overall day 50 post-DC intratracheal instillation). Bacterial burden was assessed by plating homogenized lungs on 7H10 agar plates and counting CFU. (E) Lung CD4^+^ IL-17^+^ and IFN-γ^+^ frequencies at day 50 following Mtb whole cell lysate (10 μg/ml) restimulation. (F) Correlation plots showing association between lung bacterial burden and IL-17 (left) or IFN-γ (right) responses to WCL restimulation. A linear regression was utilized to generate a best-fit line and Spearman’s correlation coefficient calculated. 4–5 mice were used for each group. Statistical significance (B-E) was determined using one-way analysis of variance correcting for multiple comparisons. * p<0.05, ** p<0.005, *** p<0.0005, **** p<0.0001, ns = not significant.

DCs loaded with Mtb antigens have been previously shown to confer better anti-mycobacterial immunity than BCG (Bacillus Calmette-Guérin) vaccination in mouse models [37, 38]. Therefore, DC-based vaccination provides a useful model to study the impact of boosting CD40-engagement on priming of antigen-specific T cell pools and on the control of Mtb infection *in vivo*. We exposed DCs to heat-killed (HK) Mtb followed by treatment with CD40LT. DCs stimulated with HK Mtb and CD40LT were equivalent to *ex vivo* assays using live Mtb (S3 Fig). Comparison groups included transfer of uninfected DCs, DCs stimulated with HK wild type Mtb or with HK *hip1* mutant Mtb. Upon transfer of antigen-loaded DCs into mouse lungs by intratracheal instillation, we assessed immune responses generated by transferred DCs by measuring the activation of endogenous CD4 T cell responses and frequencies of Th_17_ and Th_1_ cells in the lungs of mice 6 and 12 days after DC transfer. 15 days after DC transfer, we challenged mice with low-dose aerosolized Mtb. At 5 weeks post-challenge (day 50), we determined lung bacterial burden and Mtb-specific Th_1_ and Th_17_ responses (Fig 6A).

CD40LT treatment induced significantly higher frequencies of activated CD44^+^ CD4 T cells (Fig 6B) and higher frequencies of lung IL-17^+^ CD4 T cells 6 and 12 days after DC transfer (Fig 6C). IFN-γ^+^ CD4 T cell frequencies were higher on day 6 in mice receiving CD40LT-treated DCs compared to untreated Mtb-DCs, but were comparable by day 12. As expected, transfer of *hip1* mutant Mtb-stimulated DCs induced robust Th_17_ and Th_1_ responses in the lungs of mice on day 6 and day 12 post-DC transfer. Following aerosol challenge with low dose Mtb 15 days after intratracheal transfer of DCs, we assessed bacterial burden in the lungs of mice 5 weeks after challenge by plating for CFU. As shown in Fig 6D, transfer of DCs stimulated with HK Mtb resulted in significant reductions in lung bacterial burden at day 50 compared to transfer of DCs alone. Interestingly, CD40LT treatment reduced bacterial burden even further, showing that boosting CD40-CD40L interactions could overcome pathogen-mediated impairment of CD40 costimulation and promote enhanced anti-mycobacterial immunity. Notably, transfer of DCs exposed to *hip1* mutant Mtb also showed comparable reductions in bacterial burden. These results are consistent with our previous report showing that *hip1* mutant Mtb inherently induces superior DC responses compared to wild type Mtb, i.e., significantly higher induction of Th_1_- and Th_17_-polarizing cytokines, higher expression of CD40, enhanced antigen presentation and balanced Th_1_/Th_17_ responses [21]. To assess Mtb-specific Th_1_ and Th_17_ responses in the lungs of mice post-challenge, we stimulated lung cells *ex vivo* with Mtb whole cell lysate (WCL) and determined IFN-γ^+^ and IL-17^+^ CD4 T cell frequencies by flow cytometry. We found significantly enhanced Th_17_ responses in mice that intratracheally received CD40LT-treated DCs or *hip1* mutant Mtb-stimulated DCs compared to those that received Mtb-stimulated DCs. However, there was no discernible difference between the groups in terms of lung CD4 IFN-γ responses (Fig 6E). Importantly, lung IL-17 responses inversely correlated with bacterial burden, while there was no significant correlation between IFN-γ responses and lung bacterial burden (Fig 6F). Our data show that we can improve protection against Mtb challenge by overcoming Mtb-mediated impairments in CD40 costimulation.

## Discussion

The findings in this study identify the CD40-CD40L pathway as a critical mechanism for the generation of antigen-specific Th_17_ responses and highlight the importance of DC-T cell crosstalk in immunity to Mtb infection. Importantly, we provide insights into improving adaptive immunity to TB by augmenting the functions of DCs and show that exogenously engaging CD40 on DCs significantly enhances control of Mtb burden in the lungs of infected mice.

Costimulatory signals provided by antigen presenting cells such as macrophages and DCs are critical for full activation of naïve antigen-specific CD4 T cells and promote their rapid expansion into cytokine-producing effector cells, which exert their antimicrobial functions at the site of infection. While differentiation of activated CD4 T cells into IFN-γ^+^ Th_1_ subsets is relatively well understood, the molecular mechanisms underlying the generation of Th_17_ cells, particularly during Mtb infection, are less clear. Moreover, the mechanisms by which Mtb induces delayed, suboptimal T cell immunity, which enables the pathogen to successfully evade adaptive immunity and persist within the host, remain poorly understood. Several studies, including our own, have shown that Mtb impairs antigen presentation in infected DCs and dampens production of Th_17_-polarizing cytokines, such as IL-6, IL-23 and IL-1β [21, 22, 24, 39–41]. However, very little was known about the role of costimulatory pathways in driving Th_17_ development in TB prior to our study. Our work shows that innate cytokines are important for the generation of IL-17 responses (Figs 1 and 3) and is consistent with other studies showing a critical role for CD40-dependent IL-6 and IL-23 in the induction and expansion of Th_17_ cells [42–44]. Interestingly, our results show that blocking CD40-CD40L interaction with MR1 attenuates IL-17 responses to Mtb-infected DCs despite treatment of DCs with CD40LT, which suggests that optimal induction of IL-17 to Mtb infection requires CD40-CD40L interaction (Fig 3D). However, studies have shown that exogenous addition of supraphysiological levels of Th_17_-polarizing cytokines can drive *CD40*^*-/-*^ DCs to induce IL-17 [42]. Our data suggest that costimulatory interactions between Mtb-infected DCs and T cells are required for optimal generation of IL-17 responses. In addition, localization of CD4 T cells in close proximity to infected DCs is likely to be an important determinant of the type of antigen-specific CD4 T cells mobilized after infection. Recent work has demonstrated that uninfected MLN-resident DCs acquire antigen from infected lung DCs and can prime Mtb-specific CD4 T cells to produce IFN-γ [23]. It is possible that while MLN-resident DCs acquire antigen, their maturation status and costimulatory capacity may be suboptimal without Mtb infection and, thus, not amenable to generating CD4 T cell responses beyond IFN-γ. Moreover, within the Th_1_ subset, studies have shown distinct IFN-γ-producing CD4 T cells in the vasculature and parenchyma of Mtb-infected mice [45]. However, localization of Th_17_ cells within lung compartments and the role of lung-specific DC subsets in driving the polarization of Th_1_ and Th_17_ during Mtb infection are poorly understood. Our study uses bone marrow derived DCs and therefore the extent to which our experiments model *in vivo*-generated lung DCs needs to be investigated further. Overall, our data showing that CD40-CD40L interaction is required for optimal Th_17_ generation in response to Mtb and that boosting CD40-CD40L interactions augments Th_1_ and Th_17_ responses suggests that restriction of costimulatory pathways is an important virulence mechanism used by Mtb for inducing suboptimal T-helper responses that benefits the pathogen.

Our finding that exogenous induction of CD40-mediated costimulation, via CD40LT treatment, is able to elicit IL-17 production at lower concentrations of peptide stimulation (Fig 3) than by Mtb-infected DCs alone leads to an interesting speculation. In early stages of Mtb infection, low levels of antigen in the lung, combined with impaired CD40 induction on Mtb-infected DCs, likely results in suboptimal costimulation of naïve CD4 T cells and therefore suboptimal and delayed induction of Th_17_ responses. However, engaging the CD40-CD40L pathway and promoting interactions between these two molecules likely facilitates better Th_17_ generation, even when lung antigen levels are low during early stages of infection. It has been reported that higher peptide concentrations are required for inducing Th_17_ polarization compared to Th_1_ in a study that examined activation of Smarta-2 TCR-Tg T cells [42]. Efficient CD40-mediated costimulation may serve to lower the threshold for T cell activation and Th_17_ polarization, and overcome the need for high antigen loads. Interestingly, *hip1* mutant Mtb-loaded DCs induced higher Th_17_ responses compared to wild type Mtb, even without CD40LT treatment (Fig 5), and enhanced protection (Fig 6). We have previously shown that *hip1* mutant Mtb induces high levels of CD40 and Th_17_ responses [21]. Therefore, it is likely that elimination of Hip1 results in efficient CD40-dependent costimulation, and bypasses the need for exogenous engagement of the CD40-CD40L pathway. However, we do not rule out the possibility that *hip1* mutant Mtb activates alternate DC pathways that promote robust T cell immunity and further investigation into the common and exclusive immune pathways activated by CD40LT and *hip1* mutant Mtb is of interest.

Previous work by Demangel et al demonstrated that lung Th_1_ responses can be augmented by transferring BCG-infected DCs in conjunction with agonistic anti-CD40 mAb [33]. However, this approach did not significantly restrict Mtb lung burdens following challenge compared to BCG-infected DCs alone. We speculate that the use of heat killed Mtb in our study as well as the timing of the aerosol challenge at 2 weeks after intratracheal instillation of DCs (in contrast to 2 days post-DC transfer in the Demangel et al study) likely established higher frequencies of antigen-specific Th_17_ and Th_1_ precursors, leading to better control of Mtb. Additionally, recent work by Griffiths et al showed that mice vaccinated with BCG followed by intratracheal delivery of Ag85B peptide loaded DCs, one day before and four days after challenge with Mtb HN878, had enhanced bacterial control [46]. Interestingly, they achieved similar reductions in bacterial burden after administration of TLR-9 and CD40 agonists together with Ag85B peptide and also showed higher levels of lung IFN-γ and IL-17 responses. The study by Griffiths et al complements our results, which provide mechanistic evidence that the CD40-CD40L pathway is critical for the generation of Mtb-specific lung Th_17_ responses. While IL-17 responses appear to be required for resistance against infection with the hypervirulent Mtb HN878 strain, IL-17 may also be important for generating efficacious vaccine-induced immunity. Our data show an association between enhanced IL-17 responses and lower bacterial burden after aerosol Mtb challenge (Fig 6F), but do not directly link Th_17_ responses with increased protection. While we have demonstrated that engaging CD40 on DCs confers enhanced Th_17_ responses in the lungs in a setting of mucosal DC transfer, we also observed augmented Th_1_ responses *in vivo* prior to challenge (Figs 5 and 6). Therefore, our data demonstrate that CD40 engagement on DCs improves adaptive immunity to TB, likely due to induction of a balanced Th_1_/Th_17_ response. Although we have not shown that the Th_17_ cells generated in the lung following transfer of DCs stimulated with HK Mtb + CD40LT or HK *hip1* mutant Mtb are directly responsible for the increased protection seen in Fig 6, our studies provide a platform to further investigate the potential of designing vaccination strategies that overcome Mtb immune evasion, either by augmenting CD40 costimulation and/or deletion of immunomodulatory factors such as *hip1* (in BCG or other live attenuated Mtb vaccine strains) that impair DC functions.

Our studies on understanding the role of CD40 costimulation in Th_17_ responses significantly extend our understanding of the CD40-CD40L pathway during infection, as previous investigations studying this pathway in TB as well as in other infections have focused on Th_1_ responses. CD40 has been shown to promote Th_1_ responses by synergizing with TLR signaling to induce high levels of IL-12 production from antigen presenting cells in several infections [33, 47, 48]. While our own data show that *CD40*^*-/-*^ DCs and CD40LT-treated DCs infected with Mtb do not affect IFN-γ responses in a closed system *in vitro* (Figs 1 and 3), treatment of Mtb-infected DCs with CD40LT does augment IFN-γ responses in the lungs 6 days after intratracheal instillation of DCs (Figs 5 and 6), suggesting that engaging the CD40-CD40L pathway enhances both Th_1_ and Th_17_ responses *in vivo* and may lead to a more balanced Th_1_/Th_17_ immunity to TB. Engagement of CD40 is not uniquely important for Th_17_ generation, as previous investigations on the role of CD40 in mycobacterial diseases have supported the importance of CD40 in the amplification of Th_1_ responses. *CD40*^*-/-*^ mice were shown to be susceptible to aerosol infection with Mtb due to a defective Th_1_ response [28], but *CD40lg*^*-/-*^ mice were reported to be resistant to Mtb infection and capable of establishing Th_1_ immunity [28, 49]. Together with our data showing that Mtb poorly induces CD40 expression on infected DCs [21], these studies suggest that, while CD40L may be dispensable for generating Th_1_ responses that control bacterial burden, engaging the CD40-CD40L pathway may be important for generating balanced Th_1_/Th_17_ responses that may better control Mtb infection. Moreover, while IL-17 responses were not examined in those studies, mucosal Th_17_ cells are also likely to contribute to controlling Mtb in *CD40*^*-/-*^ mice *in vivo;* this may be dependent on antigen load as the reported susceptibility of *CD40*^*-/-*^ mice disappeared after high dose aerosol challenge [28]. Our work showing that promoting CD40-CD40L interaction augments early Th_17_ responses in the lung (Figs 5 and 6) is consistent with several previous reports showing an important role for Th_17_ cells in protection at mucosal surfaces such as in the lung and intestine [18, 19, 50, 51]. In TB, it has been suggested that Th_17_ cells in the lung may act directly on infected cells or by recruiting additional immune cells, such as IFN-γ^+^ Th_1_, to combat infection. Notably, in Figs 5 and 6, we show that intratracheal instillation of Mtb-infected DCs treated with CD40LT is associated with an earlier IFN-γ response in the lungs compared to Mtb-DC, which supports the idea that induction of early antigen-specific Th_17_ can serve to recruit antigen-specific Th_1_ cells. Our work highlights the importance of augmenting DC costimulation in order to improve adaptive immunity to TB and provides evidence that specifically augmenting DCs through CD40 can enhance antigen-specific mucosal immunity.

The generation of robust antigen-specific immunity that goes beyond IFN-γ-producing Th_1_ responses is an important consideration for vaccines and host-directed therapeutics for TB. The IL-12/STAT-1/IFN-γ axis is important for the control of Mtb, but robust induction of IFN-γ alone does not correlate with enhanced protection against developing TB disease in a variety of vaccine trials [6, 7], and there is mounting evidence for IFN-γ independent and Th_17_-mediated mechanisms of Mtb control [9, 52, 53]. In fact, recent work in mice has demonstrated that IFN-γ plays a more important role in control of bacterial burden at extra-pulmonary sites such as the spleen and must be restrained to prevent lung pathology [54]. In humans, bi-allelic mutations in *RORC*, leading to abrogated IL-17 responses, is associated with susceptibility to mycobacteria, suggesting a role for IL-17 responses in human TB [55]. In addition, the emerging importance of mucosal Th_17_ responses in protective and vaccine-induced immunity to Mtb [18, 19, 51] highlights the need to design and evaluate candidate vaccines that induce robust early Th_17_ responses. It is important to keep in mind, however, that unbalanced production of IL-17 can be pathogenic [56]. Over-exuberant induction of IL-17 at non-mucosal sites via repeated subcutaneous BCG exposure can lead to worsening of disease [57] and damaging neutrophilia, while IFN-γ receptor signaling limits excessive Th_17_-mediated neutrophilia [58]. In this context, future studies aimed at augmenting CD40 costimulation would benefit from studying how augmenting this pathway impacts neutrophil responses.

In summary, our studies demonstrate a novel role for CD40 costimulation in generating Th_17_ responses in TB and show that augmenting the CD40-CD40L pathway, either through DC-targeted strategies or deletion of immune-evasion genes in the pathogen, can bolster adaptive immunity in TB. Our results indicate that targeting DC costimulatory pathways in the context of subunit vaccines or live attenuated vaccines represents a novel strategy to induce balanced Th_1_/Th_17_ immunity and improve control of Mtb infection.

## Material and methods

### Ethics statement

All experiments using animals or tissue derived from animals were approved by the Institutional Animal Care and Use Committee (IACUC) at Emory University (Protocol number YER-2003476-060919GN). Experiments were carried out in strict accordance with the recommendations in the Guide for the Care and Use of Laboratory Animals of the National Institutes of Health.

### Bacterial strains

*Mtb* H37Rv was grown as described previously [21, 40]. Briefly, bacteria were grown at 37°C in Middlebrook 7H9 broth or 7H10 agar supplemented with 10% oleic acid-albumin-dextrose-catalase (OADC) (Becton Dickinson, Franklin Lakes, NJ), 0.5% glycerol, and 0.05% Tween 80 (for broth), with the addition of 25 μg/ml kanamycin (Sigma-Aldrich, St. Louis, MO) for *hip1* mutant Mtb. For heat inactivation, bacterial stocks in 7H9 were grown to midlog phase, sonicated, washed twice with PBS and inactivated at 80°C for 2 hours.

### Mice

All mice were housed under specific pathogen-free conditions in filter-top cages within the vivarium at the Yerkes National Primate Center, Emory University, and provided with sterile water and food ad libitum. C57BL/6, and C57BL/6 CD45.1^+^ congenic mice, *CD80*^*-/-*^*CD86*^*-/-*^, and *CD40*^*-/-*^ mice were purchased from The Jackson Laboratory. OT-II TCR Tg mice specific for OVA_323–339_ peptide were obtained from Dr. Bali Pulendran (originally generated in the laboratory of Dr. F. Carbone, University of Melbourne), and TCR-Tg mice specific for early secreted antigenic target 6 (ESAT-6)_1–20_/I-A^b^ epitope were obtained from Dr. Andrea Cooper (Trudeau Institute) and were bred at the Yerkes animal facility. *CD40lg*^*-/-*^ x OT-II Tg mice were obtained from Dr. Mandy Ford (Emory University) and bred at the Yerkes animal facility.

### Generation of bone marrow derived dendritic cells

For generating murine bone marrow derived DCs, bone marrow cells from indicated strains of mice were flushed from excised femurs and tibias and grown in RPMI 1640 medium (Lonza, Walkersville, MD) supplemented with 10% heat-inactivated FBS (HyClone, Logan, UT), 2 mM glutamine, 1x β-mercaptoethanol, 10 mM HEPES, 1 mM sodium pyruvate, 1x nonessential amino acids, and 20 ng/ml murine recombinant GM-CSF (R&D Systems, Minneapolis, MN). Incubations were carried out at 37°C with 5% CO_2_. Fresh medium with GM-CSF (20ng/ml) was added on days 3 and 6, and cells were used on day 8 for all experiments. We routinely obtained >85% CD11c^+^ CD11b^+^ MHCII^+^ cell purity by flow cytometry. DCs were further purified using CD11c microbead kits as per the manufacturer’s instructions (Miltenyi Biotec, Auburn, CA).

### Mtb infection of DCs

3x10^5^ CD11c-purified bone marrow derived DCs were plated onto 24-well plates overnight prior to infection. For live infections, bacteria were filtered through 5 μm filters, resuspended in complete medium, and sonicated twice for 5 seconds before addition to the adherent monolayers. Bacteria were used for infection (in triplicate) at a multiplicity of infection (MOI) of 10 or as indicated. Infection of DCs was carried out for 4 hours, after which monolayers were incubated with amikacin (200 μg/ml; Sigma-Aldrich) for 45 minutes to kill extracellular bacteria and then washed four times with PBS before incubating in complete medium. To determine bacterial input, a set of wells was lysed in PBS containing 0.5% Triton X-100 and plated onto 7H10 agar plates for CFU enumeration after 21 days. For stimulation of DCs with heat killed Mtb, DC were exposed to heat-killed Mtb at an MOI of 10 in complete medium as determined by CFU enumerated from bacterial stocks prior to heat killing. Uninfected DCs were used as controls for each experiment. For some experiments, DCs were treated with multimeric CD40LT reagent (Adipogen) concurrent with infection or stimulation. Cell free supernatants were collected after 24 hours to assay for cytokines: IL-12p40, IL-12p70, IL-6, IL-1β (BD OptEIA, San Jose, CA) and IL-23 (Biolegend, San Diego, CA) by ELISA according to manufacturers’ instructions.

### DC-T cell co-culture assays

CD4 T cells were purified from single-cell suspensions of spleen and lymph nodes from 6–8 week old transgenic mice (Naïve CD4 negative selection kit, Stemcell Technologies) of the indicated strain. Purified CD4 T cells show ≥ 99% purity by FACS analysis. DCs were incubated with 10 μg/ml (or as indicated) of OVA_323–339_ or ESAT-6_1−20_ peptide for 6 hours, washed with PBS, and infected with Mtb with or without CD40LT for 24 hours. DCs were then washed twice with PBS and co-cultured with antigen-specific CD4 T cells to achieve a 1:4 DC:T cell ratio for 72 hours. Cell free supernatants collected from co-cultured cells were analyzed for IFN-γ (Mabtech, Cincinnati, OH), IL-17 (ELISA Ready-Set-Go, eBioscience), and IL-2 (BD Biosciences) by ELISA according to the manufacturers’ instructions.

### Blockade of CD40-CD40L pathway

2x10^4^ CD11c-purified DCs were seeded in 96-well plates overnight, pulsed with relevant peptide and treated with the indicated conditions for 24 hours. Afterwards, purified antigen-specific CD4 T cells were incubated with 20 μg/ml anti-CD40L (clone MR1) blocking antibody and co-cultured with DCs to achieve a 1:10 DC:T cell ratio. Co-cultured cells were incubated at 37°C with 5% CO_2_ for 72 hours prior to harvest of supernatants for ELISAs.

### Intratracheal instillation of DCs and tissue harvest

CD11c-purified DCs were stimulated with indicated conditions for 24 hours. DCs were then washed twice and resuspended in sterile PBS at 1x10^6^/50 ul and injected intratracheally into isoflurane-anesthetized mice. For some experiments, recipient mice (CD45.1^+^) received purified 1x10^6^ ESAT-6_1−20_ TCR-Tg CD4 T cells (CD45.2^+^) one day prior to DC instillation by tail-vein injection. 6 and 12 days post-intratracheal instillation, lungs and mediastinal lymph nodes were harvested. Lungs were digested with 1 mg/ml collagenase D (Worthington) at 37°C for 30 min. For some experiments, the upper right lobe of the lung was used for determining CFU and the rest of the lung was used for cellular assays. Homogenized single-cell lung suspensions were obtained through mechanical disruption and filtered through a 70-μm cell strainer (BD Biosciences), treated with RBC lysis buffer for 3–5 min, and washed twice with cell culture media. Cells were counted and used to set up stimulations for intracellular cytokine staining and flow cytometry. **S**ingle cell suspensions were stimulated with media, ESAT-6_1−20_ (10 μg/ml), Mtb whole cell lysate (10 μg/ml), or PMA (80 ng/ml) and ionomycin (500 ng/ml) as indicated. BFA (5 μg/ml) and monensin (1:1500) were added to the stimulated cells after 1.5 hours and cells were cultured for an additional 4.5 hours, or 16 hours for whole cell lysate stimulations, and then stained for flow cytometry.

### Flow cytometry

Live cells were discriminated by a live/dead fixable aqua dead cell stain (Molecular Probes). For staining DCs, murine anti-CD11c PE-Cy7 (clone N418, eBioscience), anti-CD11b APC-Cy7 (clone M1/70, Biolegend), anti-CD40 PE-Cy5 (clone 1C10, eBioscience), anti-CD86 APC (clone GL1, eBioscience), and anti–MHC II PE (clone M5/114.15.2, BD) were utilized. For staining T cells, murine anti-CD3 V450 (clone 500A2, BD), anti-CD4 Alexa700 (clone RM4-5, BD), anti-CD8 PerCP (clone 53–6.7, BD), anti-TCR γδ BV605 (clone GL3, Biolegend), anti-CD44 APC-Cy7 (clone IM7, BD), anti-CD45.1 BV785 (clone A20, BioLegend), and anti-CD45.2 BV650 (clone 104, BioLegend) were utilized to stain for surface markers. Murine anti-RORγt PE (clone B2D, eBioscience), anti-TNFa PE-Cy7 (clone MP6-XT22, BD), anti-IFN-γ APC (clone XMG1.2, eBioscience), anti-IL-2 FITC (clone JES6-5H4, BD), and anti-IL-17 PE-CF594 (clone TC11-18H10, BD) were stained intracellularly with the BD Cytofix/Cytoperm or BD Transcription Factor kit as per manufacturer’s instructions. Staining for cell-surface markers was done by resuspending ∼1-2x10^6^ cells in 100 ml PBS with 2% FBS containing the antibody mixture at 4°C for 30 min and then washing with PBS containing 2% FBS. Data were immediately acquired using an LSRII flow cytometer (BD Biosciences). Data were analyzed with FlowJo software (FlowJo LLC, Ashland, OR).

### Aerogenic infection of mice with Mtb

Mtb H37Rv was grown to OD_600_ of ∼0.6–0.8, washed two times in 1× PBS. 1-ml aliquots were frozen at −80°C and used for infection after thawing. Single-cell suspensions of these aliquots were used to deliver ∼100 CFU into 8–10 week old C57BL/6J mice using an aerosol apparatus manufactured by In-Tox Products (Moriarty, NM). Bacterial burden was estimated by plating serial dilutions of the lung homogenates on 7H10 agar plates on day 1 (for entry) or as indicated. CFU was enumerated after 21 days.

### Statistical analyses

The statistical significance of data was analyzed using the Student’s unpaired t-test for comparisons between two groups or one-way analysis of variance (ANOVA) with a Tukey posttest correction for multiple comparisons for analysis of two or more groups (GraphPad Prism 6.0h). In order to calculate correlation, a linear regression was utilized to generate a best-fit line and Spearman’s correlation coefficient calculated (GraphPad Prism 6.0h). Data are shown as mean **±**S.D. of one representative experiment from multiple independent experiments.

## Supporting information

S1 Fig CD40L is required for IFN-γ and IL-2 responses from antigen-specific CD4 T cells *in vitro*. DCs from C57BL/6 (B6) were pulsed with OVA_323-339_ at 10 μg/ml and infected with Mtb for 24 hours followed by co-culture with purified OT-II or *CD40lg*^*-/-*^ x OT-II TCR-Tg CD4 T cells. Cell-free supernatants were collected after 72 hours and assessed for the indicated cytokines by ELISA. Values are presented as mean ± SD. Statistical significance was determined using a 2-tailed unpaired T test. * p<0.05.
(TIFF)

S2 Fig CD40-CD40L interaction can be blocked using non-agonistic anti-CD40L antibody MR1. To determine optimal concentrations of blocking antibody, 1x10^6^ splenocytes from OT-II TCR-Tg mice were plated with 5 μg/ml anti-CD16/32 (Fc Block) and pulsed with 10 μg/ml OVA_323-339_ peptide for 6 hours in the presence or absence of non-agonistic anti-CD40L antibody (clone MR1) at the indicated concentrations. After 6 hours, PE-conjugated anti-CD40L antibody (clone MR1, 1:100) was spiked into the sample and left in the dark at 37°C for 18 hours. Cells were then washed, stained for viability, CD3 and CD4, and acquired immediately. Representative flow plots of recovered CD40L expression on live CD3^+^ cells are shown demonstrating titratable blockade of CD40L by MR1.
(TIF)

S3 Fig CD40 engagement enhances cytokine production from DCs exposed to heat-killed Mtb. B6 DCs were left uninfected or exposed to heat-killed Mtb in the presence or absence of 1 μg/ml multimeric CD40LT reagent (CD40LT) for 24 hours. Cell-free supernatants were collected after 24 hours and the indicated innate cytokines were measured by ELISA. Data are representative of 3 independent experiments. Values are presented as mean ± SD. Statistical significance was determined using a 2-tailed unpaired T-test. * p<0.05.
(TIFF)
