# Supplementary figures and images for: Engaging the CD40-CD40L pathway augments T-helper cell responses and improves control of Mycobacterium tuberculosis infection
Source: PLoS Pathog. 2017 Aug 2;13(8):e1006530. doi: 10.1371/journal.ppat.1006530 (PMC5540402; doi:10.1371/journal.ppat.1006530)

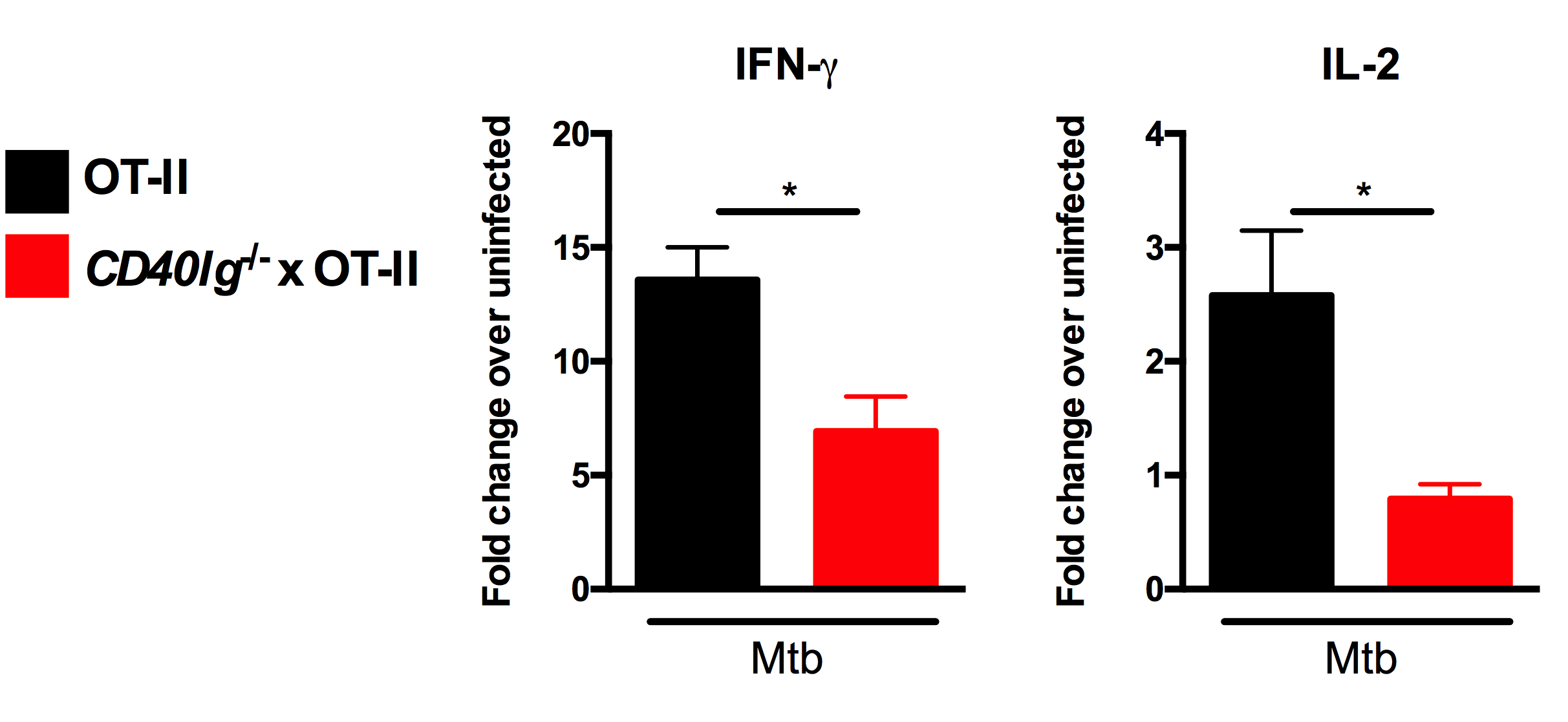

Supplement: S1 Fig — DCs from C57BL/6 (B6) were pulsed with OVA323-339 at 10 μg/ml and infected with Mtb for 24 hours followed by co-culture with purified OT-II or CD40lg-/- x OT-II TCR-Tg CD4 T cells. Cell-free supernatants were collected after 72 hours and assessed for the indicated cytokines by ELISA. Values are presented as mean ± SD. Statistical significance was determined using a 2-tailed unpaired T test. * p<0.05. (TIFF) [file ppat.1006530.s001.tiff]

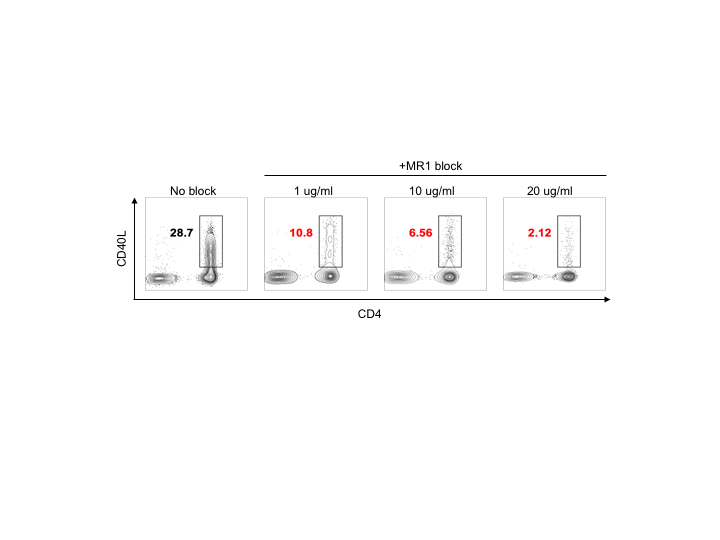

Supplement: S2 Fig — To determine optimal concentrations of blocking antibody, 1x10^6^ splenocytes from OT-II TCR-Tg mice were plated with 5 μg/ml anti-CD16/32 (Fc Block) and pulsed with 10 μg/ml OVA323-339 peptide for 6 hours in the presence or absence of non-agonistic anti-CD40L antibody (clone MR1) at the indicated concentrations. After 6 hours, PE-conjugated anti-CD40L antibody (clone MR1, 1:100) was spiked into the sample and left in the dark at 37°C for 18 hours. Cells were then washed, stained for viability, CD3 and CD4, and acquired immediately. Representative flow plots of recovered CD40L expression on live CD3+ cells are shown demonstrating titratable blockade of CD40L by MR1. (TIF) [file ppat.1006530.s002.tif]

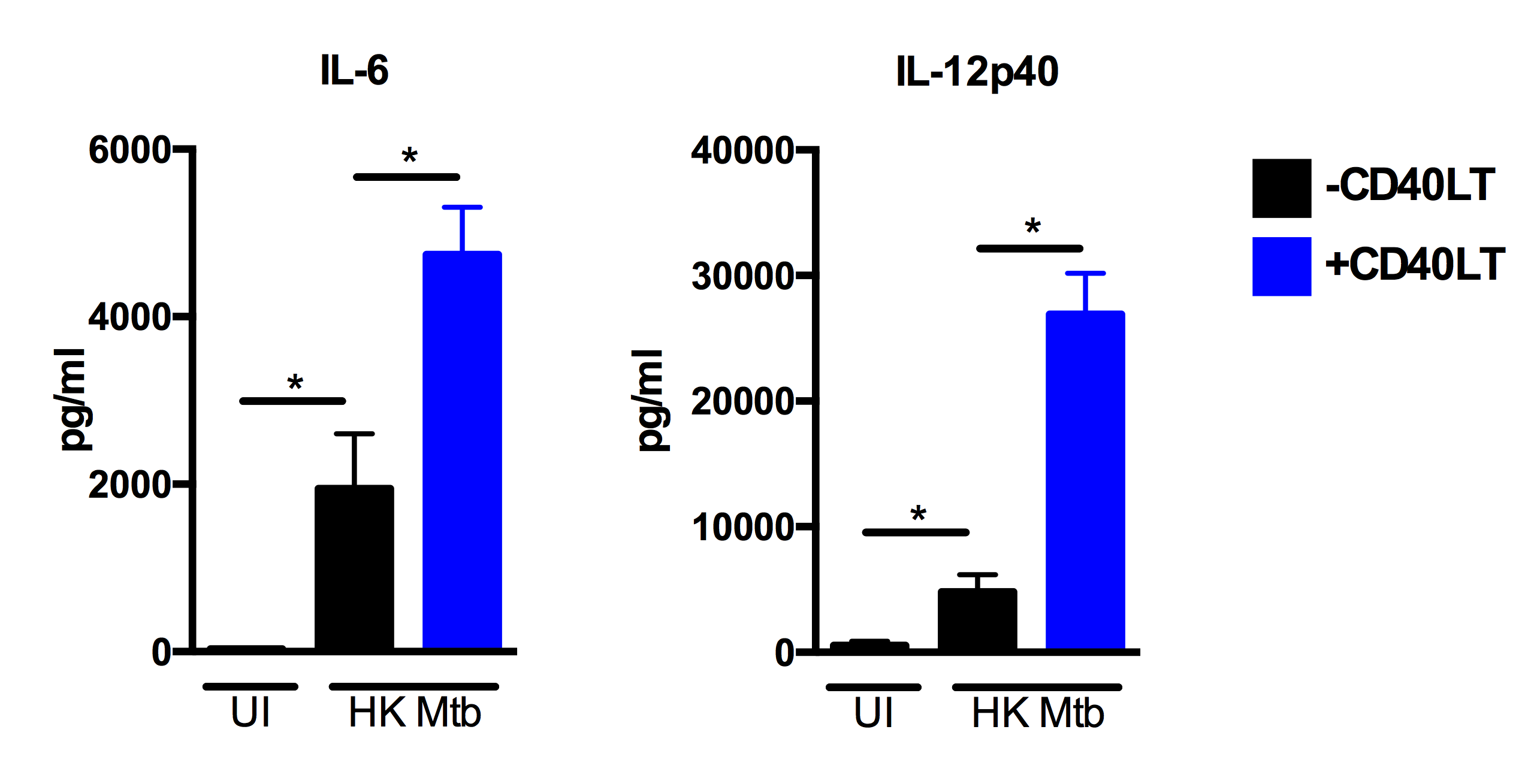

Supplement: S3 Fig — B6 DCs were left uninfected or exposed to heat-killed Mtb in the presence or absence of 1 μg/ml multimeric CD40LT reagent (CD40LT) for 24 hours. Cell-free supernatants were collected after 24 hours and the indicated innate cytokines were measured by ELISA. Data are representative of 3 independent experiments. Values are presented as mean ± SD. Statistical significance was determined using a 2-tailed unpaired T-test. * p<0.05. (TIFF) [file ppat.1006530.s003.tiff]
